# Can premium differentiation counteract adverse selection in the Dutch supplementary health insurance? A simulation study

**DOI:** 10.1007/s10198-017-0918-2

**Published:** 2017-07-31

**Authors:** K. P. M. van Winssen, R. C. van Kleef, W. P. M. M. van de Ven

**Affiliations:** Institute of Health Policy and Management, Erasmus University Rotterdam, Burgemeester Oudlaan 50, 3062 PA Rotterdam, The Netherlands

**Keywords:** Adverse selection, Adverse selection spiral, Death spiral, Health insurance, Premium, Premium differentiation, Supplementary insurance, C15, D12, D82, G22, I11, I13

## Abstract

Most health insurers in the Netherlands apply community-rating and open enrolment for supplementary health insurance, although it is offered at a free market. Theoretically, this should result in adverse selection. There are four indications that adverse selection indeed has started to occur on the Dutch supplementary insurance market. The goal of this paper is to analyze whether premium differentiation would be able to counteract adverse selection. We do this by simulating the uptake and premium development of supplementary insurance over 25 years using data on healthcare expenses and background characteristics from 110,261 insured. For the simulation of adverse selection, it is assumed that only insured for whom supplementary insurance is expected not to be beneficial will consider opting out of the insurance. Therefore, we calculate for each insured the financial profitability (by making assumptions about the consumer’s expected claims and the premium set by the insurer), the individual’s risk attitude and the probability to opt out or opt in. The simulation results show that adverse selection might result in a substantial decline in insurance uptake. Additionally, the simulations show that if insurers were to differentiate their premium to 28 age and gender groups, adverse selection could be modestly counteracted. Finally, this paper shows that if insurers would apply highly refined risk-rating, adverse selection for this type of supplementary insurance could be counteracted completely.

## Introduction

By the Health Insurance Act (2006), Dutch inhabitants are obliged to take out basic health insurance from a private health insurer of their choice, which covers a basic benefit package determined by the government. For healthcare services not covered by basic insurance, insured can voluntarily purchase supplementary health insurance. Healthcare services covered by supplementary insurance include, among others, dental care for adults, physiotherapy, durable medical equipment, alternative medicines, pharmaceuticals, care consumed in a foreign country, orthodontics and maternity care, as far as these benefits are not covered by basic health insurance. Contrary to basic insurance, supplementary insurance in the Netherlands is offered at a free market. Instead of the requirements of community-rating and open enrolment that hold for the basic health insurance, insurers on the supplementary insurance market are free to apply risk-rating and selective underwriting. Nevertheless, as a result of societal pressure, many Dutch insurers do still apply community-rating and open enrolment. Theory predicts that these circumstances lead to adverse selection [35]. This paper focuses on adverse selection in the Dutch supplementary health insurance market and the potential of premium differentiation to counteract it.

Adverse selection refers to the tendency that, within each premium risk group, high-risk individuals have a stronger incentive to buy supplementary insurance or to extend their coverage compared to low-risk individuals. It arises as a result of asymmetric information^1^ between the insured and the insurer [5, 10]. More specifically, the (applicant) insured has information regarding his risk that the insurer does not have, is not willing to use or is not allowed to use for risk rating or selective underwriting [1, 26, 27]. Two conditions are necessary for adverse selection to arise (e.g., [2, 4, 23, 35, 47]. Firstly, insured need to be able to better forecast their expected healthcare expenses than is reflected within the premium. Secondly, this forecast needs to affect the demand for insurance. As a result of adverse selection, the insurer’s profit is less than anticipated and the premium of supplementary insurance has to be increased. In the next year, this premium increase provides an incentive for low-risk individuals (within their premium risk group) to leave the supplementary insurance policy or to reduce their coverage. This continuing process may lead to a so-called adverse selection or death spiral.^2^ Adverse selection may have considerable consequences, since it might cause a competitive health insurance market to become unstable [35]. In stable markets, both low-risk and high-risk individuals purchase the insurance policy especially designed for them. In unstable markets, due to adverse selection, individuals might select the wrong health policy (i.e., the policy that is not optimal given their expected healthcare expenses and preferences) [9]. Additionally, insurers could, in an attempt to counteract adverse selection, manipulate their offerings to deter the sick and attract the healthy insured [9]. These manipulations might impose welfare losses since they deny both low-risk and high-risk individuals the coverage they would like most.

Historically, almost all Dutch individuals purchased supplementary health insurance [46]. There are, however, four indications that adverse selection has started to occur in the Dutch supplementary health insurance. Firstly, the percentage of individuals with supplementary insurance decreased from 93% in 2006 to 84% in 2016 [46]. Additional research shows that the majority of individuals without supplementary insurance (i.e., 72% in 2014) did not purchase supplementary insurance because they expected not to need the healthcare services covered [33]. Secondly, individuals with a supplementary insurance take out insurance policies with less comprehensive coverage than before [13, 45]. In 2011, for instance, 75% of the insured took out coverage for dental care, while in 2012 this reduced to 65%. Additionally, 66% of the insured took out coverage for physiotherapy in 2011, while this reduced to 49% in 2012 [13]. Thirdly, insurers’ total technical result on supplementary insurance policies reduced substantially, from 321 million in 2008 to 33 million in 2014 [45]. Fourthly, more and more insurers stop offering supplementary insurance policies providing coverage for healthcare services mostly used by high-risk individuals (e.g., unlimited coverage for physiotherapy), while premiums for policies that do offer this coverage increased considerably [49]. There are several potential explanations for the increase of adverse selection, such as changes in an individual’s financial conditions due to for instance economic crisis, changes in the basic benefit package (e.g., fewer healthcare services reimbursed or lower maximum reimbursement levels) and changes in the entitlement to reimbursement. Additionally, two developments may lead to a further increase of adverse selection in the upcoming years. Firstly, there is an increase in media attention that urges insured to critically review their need of purchasing supplementary health insurance to reduce unnecessary coverage and to search for the lowest premiums (e.g., [7, 16–18, 20, 21, 29, 31]). This may potentially encourage healthy individuals to opt out of the supplementary insurance, causing an increase in adverse selection. Secondly, it might be expected that insurers over the last few years have tried to limit the increase in premium for supplementary health insurance by reducing their profit. However, insurers may no longer be able to do this, since the technical result on supplementary health insurances has reduced significantly [43]. This might imply that (substantial) premium increases could be expected for supplementary health insurance in the upcoming years causing an increase in adverse selection. To counteract adverse selection, insurers are allowed to apply premium differentiation,^3^ although currently only very few Dutch insurers actually do this.^4^

Against this background, this paper studies whether premium differentiation would be able to counteract adverse selection. This is studied by simulating the uptake of supplementary health insurance and the associated development in insurance premium over time using data on healthcare expenses and background characteristics from 110,261 Dutch insured. The next two sections, respectively, discuss the data and methods used for the empirical simulations. The results are presented in section four. Sections five and six, respectively, present the conclusion and provide points for discussion, directions for further research and policy implications.

## Data

For the simulations of adverse selection over time and the potential of premium differentiation to counteract adverse selection, we use individual-level information on healthcare expenditure and risk characteristics from the Achmea Health Database. The dataset contains 110,261 individuals who had the same supplementary health insurance policy during the entire period 2006–2011. Their supplementary insurance covers dental expenses and healthcare services not covered by basic health insurance (e.g., physiotherapy, alternative medicine, care consumed in a foreign country, etc.). In 2011, the annual premium for this policy was almost €500, while the average premium for supplementary insurance in the Netherlands in that year was little over €300 [45]. In the Netherlands, children can be insured on the supplementary health insurance policy of one of the parents without any additional costs. This means that children do not actually have a direct demand for supplementary insurance. Therefore, we only included adult insured (i.e., 18 years or older on January 1, 2006) into our analyses.

The Achmea Health Database contains administrative data from a large Dutch health insurer operating mainly in the western and eastern parts of the Netherlands. The data contain individual-level information on insurance claims,^5^ both for basic insurance and supplementary insurance, aggregated at and categorized into the following thirteen types of healthcare services: GP-care, pharmacy, inpatient care, hospital admissions, outpatient care, dental care, maternity care, durable medical equipment, physiotherapy, mental care, care consumed in a foreign country, alternative medicines and glasses. Furthermore, the database includes an encrypted ID number and (per year) information on the year of birth, sex, ethnicity, degree of urbanization in the residential area and in which Pharmacy-based Cost Group (PCG) and/or Diagnoses-based Cost Group (DCG)^6^ the insured is classified for the risk equalization scheme.

Appendix 1 provides an overview of some background characteristics of the Dutch population and compares these to the characteristics of our sample. It shows that more insured in the data are classified into a PCG and that the data includes a smaller share of insured up to the age of 40 compared to the entire Dutch population. This might be a result of the adverse selection that has already taken place for this supplementary insurance over the course of time. It will not affect our results concerning the uptake of supplementary insurance since the simulations only take into account who is relatively healthy or unhealthy compared to the entire sample; i.e., is the individual a low-risk or high-risk within the relevant premium risk group (see “Methods”).

## Methods

### Descriptive statistics

In the data, the average healthcare expenses under supplementary insurance in 2011 for the selected 110,261 insured are €221. Table 1 provides an overview of the average healthcare expenses under supplementary insurance in 2011 broken down into deciles. It shows that many insured (i.e., almost 30%) have no healthcare expenses under supplementary insurance at all. On the other hand, it shows that the top 10% has substantial average healthcare expenses under supplementary insurance. The five insured with the highest healthcare expenses under supplementary insurance in 2011 had healthcare expenses of €7421, €10,659, €14,532, €26,293, and €33,250. Furthermore, 3.36% of the insured in the database had healthcare expenses under supplementary insurance larger than €1000 in 2011 and only 0.01% of the insured in the database had healthcare expenses under supplementary insurance larger than €5000.

**Table 1:** Average actual health claims under supplementary insurance (SHI) in 2011 broken down into deciles

Decile	Percentage insured^a^	Average claims under SHI in 2011 (€)
1	10	0
2	19.3	0
3	0.5	13.58^b^
4	10.1	44.71
5	10.5	101.18
6	9.5	147.70
7	8.8	213.06
8	11.2	271.98
9	10.0	429.37
10	10.0	996.32

^a^The fact that the deciles do not consist of 10% of the total number of insured is caused by the reimbursement limits. As a result, a substantial number of individuals have roughly the same amount of claims, which causes them to end up in the same decile

^b^Notice that due to the fact that the vast majority of the group of insured belonging to the third decile actually have zero healthcare claims, the average claims presented here are of the small group of insured belonging to the third decile who actually had claims

### Simulation process

**Fig. 1:**
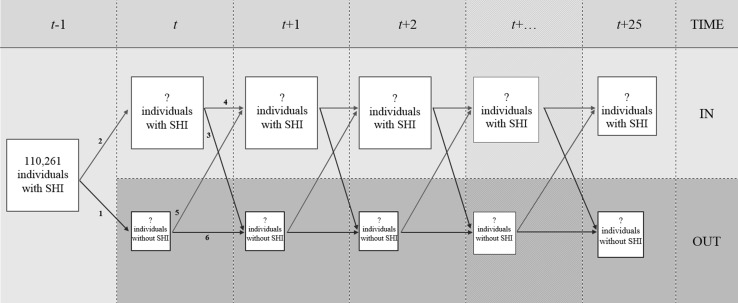
The simulation process

Using the above-mentioned data, we will simulate the uptake and premium development of supplementary health insurance over time and study the potential of premium differentiation to counteract adverse selection. In general, we will simulate per year who takes out a supplementary insurance and who does not^7^ (see Fig. 1). We use year *t* − 1 (i.e., 2007) as our base year in which 100% of the insured still have their supplementary health insurance policy. From that year, we start the simulation process and study the effect of adverse selection on the premium. In year *t*, two flows of insured are simulated. Firstly, a share of the insured for whom purchasing supplementary insurance is expected not to be financially beneficial will opt out due to adverse selection (i.e., line #1 in Fig. 1). Secondly, the remainder of this group will not opt out of the policy and a share of insured is expected to benefit from purchasing supplementary insurance and will therefore keep the policy (i.e., line #2 in Fig. 1). This process leaves us with a group of insured with and without supplementary health insurance in year *t*. The premium for year *t* + 1 is based upon the average claims of the group of insured with supplementary insurance in year *t*. As a result, four flows of insured are simulated in year *t* + 1. Firstly, for a share of insured who have supplementary insurance in year *t*, purchasing supplementary insurance is expected not to be financially beneficial in year *t* + 1 and a share of this group will opt out of the policy due to adverse selection (i.e., line #3 in Fig. 1). Secondly, the remainder of this group will not opt out of the policy and a share of insured who have supplementary insurance in year *t* is expected to benefit from purchasing supplementary health insurance in year *t* + 1 and will therefore keep the policy (i.e., line #4 in Fig. 1). Thirdly, for a share of insured who do not have supplementary insurance in year *t*, purchasing supplementary health insurance is expected to be financially beneficial in year *t* + 1, meaning that they will opt back into the insurance policy (i.e., line #5 in Fig. 1). Fourthly, for a share of insured who do not have supplementary insurance in year *t*, purchasing supplementary insurance is expected not to be financially beneficial in year *t* + 1. Therefore, they will remain out of the insurance policy (i.e., line #6 in Fig. 1). The simulation process from year *t* + 1 will be continued up to year *t* + 25 to provide insights over an extensive period of time. This is done by continuously ‘looping’ the available years in our data, meaning that we use the same 5 years (i.e., 2007–2011) over and over again to create a simulation of 25 years.^8^ Since the healthcare expenses in our data are corrected for inflation, this looping process gives no shocks in total healthcare expenses over the years but does lead to some irregularities in the distribution of healthcare expenses that will be discussed in the results section (see footnote 11).^9^

As mentioned already, we are interested in the potential of premium differentiation to counteract adverse selection. The simulation of adverse selection can be done in many different ways, such as by creating an outflow of insured based upon background characteristics (e.g., insured without any illness will opt out of the policy), based upon the lowest predicted healthcare expenses and/or based upon the number of years no claims have been filed for supplementary health insurance. In this paper, we simulate that insured for whom purchasing supplementary health insurance is not expected to be financially beneficial are subject to adverse selection and potentially opt out. Lines #1 and #3 in Fig. 1 represent this. In the Netherlands, the percentage of insured with supplementary health insurance decreased by about 1% each year over the last decade [46]. In order to simulate a continuation of this trend and study the effect on the premium, we have to make a decision regarding the probability that an insured for whom purchasing supplementary health insurance is expected not to be beneficial will opt out of the policy. Our analyses have shown that in order to simulate the continuation of adverse selection this probability must be set at 0.05. Subsequently, we simulate that from the group of insured for whom purchasing supplementary health insurance is expected not to be financially beneficial randomly 5% opts out. Note that since we select insured who opt out randomly (from the group for whom purchasing supplementary health insurance is expected not to be beneficial), we provide a lower bound of adverse selection, compared to selecting, for instance, the 5% of insured with the lowest predicted expenses. The simulation of this continuation might provide an underestimation of adverse selection in the Dutch supplementary health insurance. After all, adverse selection does not only imply that insured opt out of the policy, but it could also imply that insured reduce their coverage. This reduction of coverage is one of the indications that adverse selection has started to occur in the Dutch supplementary health insurance. Therefore, we also simulate stronger adverse selection by increasing the probability to opt out to 0.1.

### Financial profitability

A crucial parameter in our simulations concerns the financial profitability of purchasing supplementary health insurance, which is determined by the individuals’ predicted claims, the premium set by the insurer and the insured’s risk attitude. These aspects and the corresponding assumptions are discussed below.

#### Predicted claims

The amount of claims that the insured expects to have for supplementary health insurance will for a large part determine whether purchasing supplementary health insurance might be beneficial. If an insured expects no claims for supplementary insurance, he might be less inclined to purchase insurance compared to a situation in which he expects many claims for supplementary health insurance. To determine these predicted claims for each insured, several models were tested (see Appendix 2). Since all models seem to perform equally well, we use the most commonly applied GLM with a log-link and a gamma distribution [3]. The dependent variable is the total healthcare expenses under supplementary insurance in year *t*. The independent variables indicate several background characteristics that are included in the database: an age and gender interaction, classification into a PCG and/or DCG in year *t* (based upon information from year *t* − 1), degree of urbanization in the residential area, ethnicity, in which vigintile the insured was classified based upon healthcare expenses for basic insurance in year *t* − 1 and in which decile the insured was classified based upon healthcare expenses for supplementary insurance in year *t* − 1. For the years 2007–2011, we use this model to determine the predicted claims for each specific year. Table 2 shows the predicted healthcare expenses under supplementary insurance for 2011 broken down into deciles. It shows that, in the case of community-rating, the predicted healthcare claims leave substantial room for adverse selection. It additionally shows that there is potential for premium differentiation.

**Table 2:** Average predicted claims under supplementary insurance (SHI) in 2011 broken down into deciles

Decile	Percentage insured	Average predicted claims under SHI in 2011 (€)
1	10	61.06
2	10	88.37
3	10	116.89
4	10	146.04
5	10	170.66
6	10	194.07
7	10	220.16
8	10	256.59
9	10	323.81
10	10	632.83

#### Premium

Next to the predicted claims, the premium determines the financial profitability of purchasing supplementary insurance. Theoretically, the premium set by the insurer is determined as the predicted claims (as predicted by the insurer) plus a loading fee (for each premium risk group). In our data, we only have information on the premium in 2011. However, we want to simulate insurance uptake over 25 years and therefore we determine the premium as the average reimbursed claims in the prior year plus a loading fee. The loading fee for each year is based upon the average loading fee^10^ in the Netherlands in the period 2008-2011 and is 23%. This implies that the premium in year *t* + 1 is determined as the average claims (in the relevant risk group) in year *t* plus a 23% loading fee. Note that we apply a constant percentage for the loading fee over the years and over the premium risk groups, while insurers are free to (and will most likely apply) different loading fees each year and for each premium risk group depending upon their own business model.

Although many insurers do still apply community-rating, Dutch insurers are allowed to apply premium differentiation to counteract adverse selection. In order to study the potential of premium differentiation to counteract adverse selection, three modalities of premium differentiation are distinguished. Firstly, we apply premium differentiation based upon 28 age-gender classes (i.e., equal to those used in the model to predict the individual’s predicted claims). In this case, the premium the insurer sets in year *t* + 1 is based upon an OLS model based upon the group of insured who had supplementary insurance in year *t*. The dependent variable in that case is total healthcare expenses in year *t* and the independent variables are interaction terms between age and gender in year *t* − 1. Secondly, we apply premium differentiation based upon 140 age-gender-quintile classes. This means that we use the same age and gender classes but as an extra risk factor add the quintile of prior healthcare expenses. In this case, the premium the insurer sets in year *t* + 1 is based upon an OLS model with total healthcare expenses in year *t* as the dependent variable and an age, gender, and quintile of healthcare expenses of year *t* − 1. Note that for the simulation of adverse selection in year *t* + 1 (i.e., who takes out supplementary insurance and who does not), we use age, gender, and quintile of healthcare expenses of year *t*. Finally, the most complete modality of premium differentiation that we simulate is a premium in year *t* based upon the individual’s predicted claims (as described in "Predicted claims") in year *t* − 1 plus a loading fee. This modality of premium differentiation is similar to complete risk-rating the premium and fully exploits the information in our dataset.

#### Risk attitude

Next to the insured’s predicted claims and the premium set by the insurer, the insured’s risk attitude determines whether purchasing supplementary health insurance is expected to be beneficial [22, 37]. A rational risk neutral insured would purchase supplementary health insurance if his predicted claims equal or exceed the premium. So, if the premium for supplementary health insurance is, for instance, €500, a risk-neutral insured would only purchase this insurance if his predicted claims are at least €500. Insured are, however, not risk neutral regarding uncertain choices in health insurance [19]. In these situations, most insured are known to be risk averse, implying that the insured prefers a certain prospect (*x*) to any risky prospect with expected value *x*. So, a risk-averse insured is willing to pay an additional risk premium to insure himself for healthcare services covered by (supplementary) insurance. In the previous example, if the insured is for instance willing to pay a risk premium of €100, he would purchase supplementary insurance if his predicted claims plus the risk premium of €100 are at least €500. The insured’s degree of risk aversion determines the risk premium the insured is willing to pay. As risk aversion becomes larger, the risk premium the insured is willing to pay becomes larger.

We use the measure of risk aversion (*r*) developed by Pratt [28] to determine the risk premium for our simulations. Several researchers have empirically estimated this measure. Van de Ven and Van Praag [37] found an average *r*-value of 0.0067 among high-income people in the Netherlands. Marquis and Holmer [24] report *r*-values of 0.00094 and 0.00113. Finally, Van Kleef et al. [40] use *r*-values of 0.003 and 0.005 to determine the insured’s demanded compensation for opting for a voluntary deductible. Based upon the formula by Pratt [28], we use the following formula to determine the risk premium the average insured is willing to pay:

1 $${\text{RP = 0}} . 5 \times {\text{S}}^2\left( {E\left( {\overline{\text{HCE}} \left( {\text{SHI}} \right)} \right)} \right) \times r$$

where the risk premium (RP) is determined as 0.5 times the variance of the average expected claims [$\overline{\text{HCE}}$ (healthcare expenses)] under supplementary health insurance (SHI) times the risk aversion measure (*r*). Since, in our data, we have information on the variance of the supplementary insurance claims for the period 2006–2011, we determined the risk premium for the highest variance level (i.e., 28,703 in 2010) and the smallest variance level (i.e., 24,596 in 2011) using both the largest (i.e., 0.0067 [37]) and the smallest mentioned *r*-value (i.e., 0.00094 (Marquis and Holmer, 1986)). This results in, respectively, a lower bound and upper bound of the risk premium of €12 and €96. Note that the upper bound is based upon the average *r*-value as found by Van de Ven and Van Praag [37] for high-income people. They, however, show that the *r*-value differs substantially between low-income and high-income people (i.e., varying from 0.0049 for high-income people to 0.0079 for low-income people). To compensate for the variation in these results, we simulate risk aversion using a risk premium of €100. Using this risk premium, we simulate that insured with predicted claims plus a risk premium of €100 smaller than the premium (in their premium risk group) are subject to adverse selection and might potentially opt out of the supplementary health insurance. The results of the simulations are presented in the next section.

## Results

### Adverse selection

**Graph 1:**
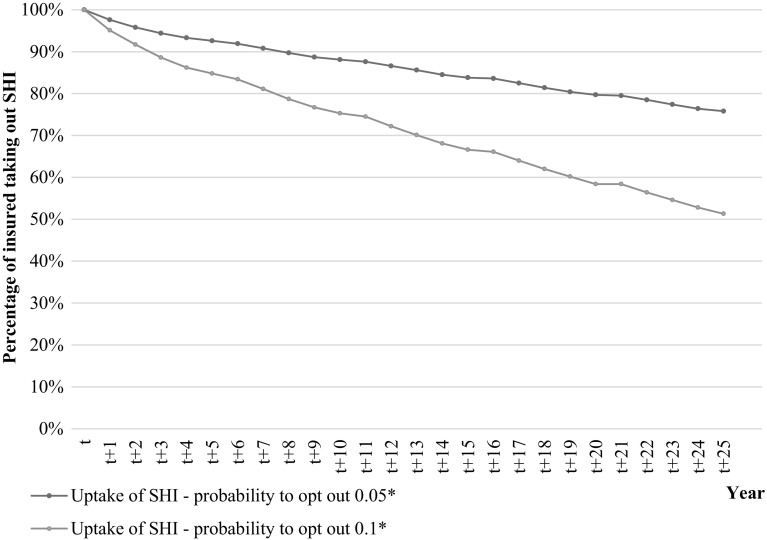
Effect of adverse selection on the uptake of supplementary health insurance (SHI). The small ‘bumps’ in the lines are caused by the fact that the group of insured becomes less healthy as time continuous. This is firstly caused by the fact that we select a group of insured who continuously take out supplementary insurance during the entire period on which the data are based, implying that they might benefit from taking out supplementary insurance because their health might be worse than the health of those insured leaving the supplementary insurance. Secondly, this might be caused by the fact that insured age as time continuous and might develop an illness. However, since we continuously use the same data sequence (i.e., 2007–2011) the health of these insured is ‘reset’ at the beginning of each new cycle. This makes them appear to be healthy again in the analyses, while they were not healthy in the year before (since that was the last year of the sequence). Note, however, that each insured’s health still corresponds to the insured’s healthcare expenses for that year. *Within the group of insured for whom purchasing supplementary insurance is expected not to be beneficial, the probability to opt out (resulting from adverse selection) is, respectively, 0.05 (*blue*/*upper line*) and 0.1 (*green*/*bottom line*) (color figure online)

Graph 1 shows the results of the simulation of the effect of adverse selection on the uptake of supplementary health insurance. The blue line indicates a continuation of adverse selection following the current trend in the Dutch supplementary health insurance, while the green line indicates stronger adverse selection (for instance because the current degree of adverse selection is underestimated since insured might have reduces their coverage instead of opting out). The blue line shows that (indeed) after 25 years, the percentage of insured taking out supplementary insurance is only 75. The green line, on the other hand, shows that after 25 years only a little more than 50% of the insured still purchase supplementary insurance.

### Premium differentiation

**Graph 2:**
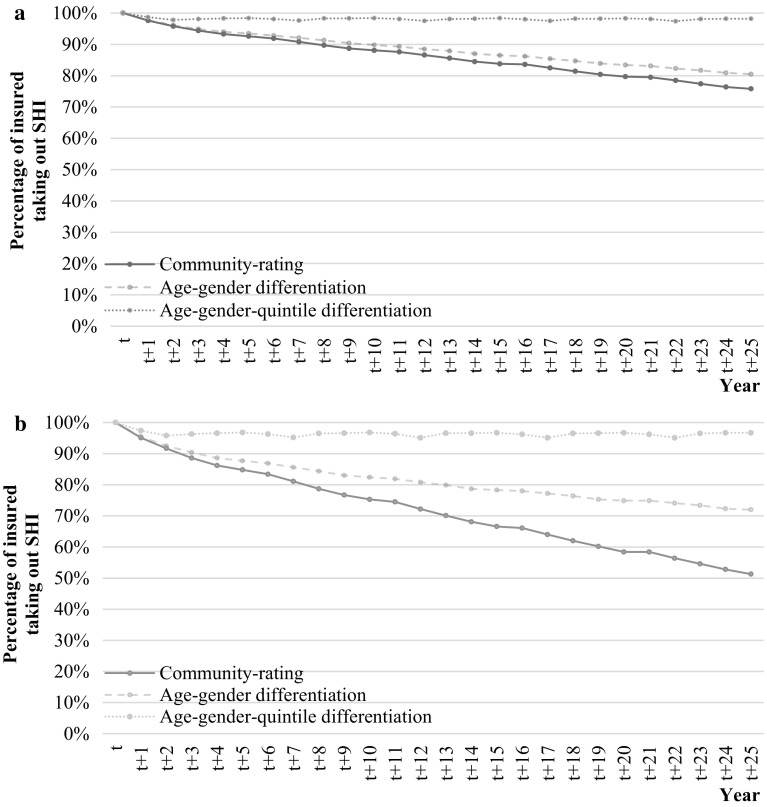
Effect of premium differentiation on the uptake of supplementary health insurance (SHI) if within the group of insured for whom purchasing supplementary insurance is not expected to be beneficial, the probability to opt out (resulting from adverse selection) is, respectively, 0.05 (**a**) and 0.1 (**b**)

Graph 2a, b show the results of the simulations of the potential of premium differentiation to counteract adverse selection, where the uninterrupted lines are equal to those in Graph 1, implying community-rating. Note that the graphs do not show the results for the complete risk-rated premium differentiation since those results were found to be more or less similar to the results of the age-gender-quintile differentiation.

The graphs show that premium differentiation to 28 different age-gender groups only counteracts adverse selection moderately. Especially in Graph 2a, this degree of premium differentiation results in an uptake of supplementary insurance over 25 years of 80% (compared to 75% in case of community-rating). In comparison, Graph 2b shows that this degree of premium differentiation is able to counteract adverse selection to an uptake of supplementary insurance over 25 years of 72% (compared to 50% in case of community-rating).

Furthermore, in Graph 2a, b premium differentiation according to 140 groups based upon age, gender, and quintile of prior healthcare expenses stabilizes the uptake of supplementary insurance at 98 and 95%, respectively.^11^ Since individuals are with this degree of premium differentiation confronted with a premium that more or less is similar to their predicted healthcare claims, purchasing supplementary health insurance is expected to be beneficial to a larger share of insured, and therefore adverse selection does hardly seem to occur, compared to the situation with community-rating.

## Conclusions

The Dutch supplementary health insurance is offered at a free market. Nevertheless, many insurers do still apply community-rating and open enrolment. Theoretically, this should result in adverse selection. There are several indications that adverse selection has started to occur on the Dutch supplementary insurance market. This paper studies the potential of premium differentiation to counteract adverse selection. In order to do so, the uptake of supplementary insurance over time is simulated using data on healthcare expenses and background characteristics from 110,261 insured. For the simulation of adverse selection, it is assumed that only insured for whom purchasing supplementary health insurance is expected not to be beneficial will consider to opt out of the supplementary insurance. Firstly, the simulation results show that as adverse selection continues in the same trend, the uptake would be 75% in 25 years, while if adverse selection would be larger, the uptake would decrease to 50% in 25 years. Secondly, the simulations show that if insurers were to differentiate their premium to 28 age-gender groups, the uptake of supplementary insurance would be either 80% in case of a continuation of adverse selection or 72% in case of stronger adverse selection. This implies that this degree of premium differentiation is only limitedly able to counteract adverse selection. Finally, the results show that in case of substantial premium differentiation, either to 140 groups based upon age, gender, and quintiles of prior healthcare expenses or an almost completely risk-rated premium, the uptake would stabilize at over 95%. This implies that with highly refined risk-rating of the premium, insurers could counteract adverse selection.

## Discussion

This section discusses the assumptions made for the empirical simulations and it presents some directions for further research related to these assumptions. Furthermore, it provides some policy implications of the results presented in this paper.

### Assumptions and further research

Regarding the empirical assumptions, we provide four points for discussion. Firstly, in this paper the insured’s decision to purchase supplementary insurance is based upon the predicted financial profitability of supplementary insurance for 1 year only. However, one could imagine that insured have more information than is reflected in our calculated predicted claims based upon information from a health insurer, for instance for planned medical care like maternity care, orthodontics, and physiotherapy. In such cases, the information asymmetry between the insured and the insurer is larger compared to the simulations in this paper, and consequently the decline in insurance uptake and the increase in premium might be steeper.

Secondly, in our simulations, the increase in the premium resulting from adverse selection only affects the size of the group for whom purchasing supplementary health insurance is expected not to be beneficial. It does not affect the probability that an insured for whom purchasing supplementary insurance is expected not to be beneficial will opt out. Our simulations therefore provide an underestimation of adverse selection. After all, one could imagine that the further away the premium is from the predicted claims (and the risk premium), the larger might be the probability that an insured would opt out since the potential financial profit of opting out becomes larger.

Thirdly, in the simulations in this paper, insurers do not apply selective underwriting. This paper simulates insurance uptake in this way since in the Netherlands all insurers have incorporated a guaranteed renewability in each supplementary health insurance [34]. This implies a guaranteed renewal of the supplementary health insurance with an equal adjustment of the premium and insurance conditions for all current insured with that specific supplementary insurance [39]. However, next to premium differentiation, another way for insurers to anticipate upon adverse selection concerns selective underwriting. In that case, insurers adjust the accepted risk to the stated premium of a certain insurance policy [39]. This could for instance be done by refusing applicants or by excluding pre-existing medical conditions from coverage for new contracts, but also for their current enrollees. However, if insurers were to apply selective underwriting, they would probably refuse high-risk individuals from purchasing their insurance policy, which decreases adverse selection, implying that only low-risk individuals can purchase supplementary health insurance. As a result, there might be a large decline in insurance uptake due to refused applicants, but thereafter the decline in insurance uptake might be less steep than simulated in this paper due to less adverse selection. Further research concerning the effect of selective underwriting on adverse selection might provide important insights.

Fourthly, we assume that the insured is willing to pay a risk premium of €100 to purchase supplementary health insurance. Although this risk premium is based upon prior research concerning risk aversion in health insurance, we are unable to state with certainty whether this risk premium captures the (Dutch) insured’s real level of risk aversion. If the insured would for instance be more risk averse—thus willing to pay a larger risk premium—than simulated within this paper, the outflow of insured would, ceteris paribus, be smaller resulting in a slower increase of the premium since there is less adverse selection.^12^ The opposite holds true in case the insured would be less risk averse than simulated within this paper. Further research into the degree of risk aversion and its effect on (supplementary) insurance uptake, premium development, and subsequently on the emergence of adverse selection is necessary.

### Policy implications

If insurers would want to anticipate upon the emergence of adverse selection or even counteract adverse selection, they need to move towards equivalence. To do so, they have three options. Firstly, they could start applying premium differentiation instead of community-rating. The results in this paper have shown that a differentiation to age and gender (which is modestly done by a few Dutch insurers) might have a modest effect on the uptake of supplementary insurance. This implies that insurers would have to use (more) refined risk-rating of the premium to be able to counteract adverse selection. In that case, however, supplementary health insurance might become unaffordable for some insured. Secondly, insurers could apply (strong) selective underwriting with which the accepted risk would be much better adjusted to the stated premium than without selective underwriting. As a result, less adverse selection could occur. This however implies that some insured will no longer be accepted for the supplementary health insurance policy they might want to purchase. Thirdly, insurers could change the design of the supplementary health insurance altogether in an attempt to counteract adverse selection. Previous research has shown that the design of the Dutch supplementary health insurance is far from optimal, since it only provides a limited reduction of financial uncertainty and provides access to already affordable healthcare services [42]. In an attempt to provide a larger welfare gain to the insured from purchasing supplementary health insurance, the design of supplementary insurance could be adjusted in a way that it does (a) provide protection against unpredictable and large financial losses (e.g., dental care after an accident), (b) has first-euro cost-sharing with an individual cap on out-of-pocket expenses (i.e., in order to reduce moral hazard and protect insured from out-of-pocket expenses they cannot afford), and (c) provides the option to save for predictable small losses such as dental check-ups (i.e., in order to make sure that insured have enough money available for these healthcare services and are not limited by liquidity constraints). Such a change in the design of the supplementary health insurance would also imply that decisions regarding the basic benefit package might become more important.

The mentioned options insurers have to counteract adverse selection are currently only very limitedly applied by insurers due to a fear of reputation loss. This shows an interesting tension on the Dutch supplementary health insurance market. On the one hand, if insurers would continue offering supplementary health insurance in the way they have done the last decade, supplementary health insurance may eventually no longer be offered due to adverse selection. On the other hand, if insurers would want to anticipate upon adverse selection, they might be compelled to apply highly refined risk-rating and selective underwriting, which might imply that supplementary insurance is no longer affordable or available for everyone. The latter strategy is not in conflict with the view of the Dutch government that solidarity in the supplementary health insurance is no goal of the government, despite the fact that the Dutch society does expect solidarity for supplementary insurance (e.g., [6, 8, 15, 30, 32]). It is therefore important to realize that, in the long run, solidarity cannot be achieved on a free competitive health insurance market.

## Appendix

### Appendix 1: Comparing the Dutch population and the sample in 2011

See Table 3.

**Table 3:** Comparing characteristics of the Dutch population and the insured within the data. For comparison reasons, children are included into this analysis

	2011	
	The Netherlands (%)	Data (%)
PCG (yes)	21.4	26.5
DCG (yes)	8.7	4.8
0–18 years^a^	23.5	16.2
19–40 years	25.0	20.5
41–65 years	35.9	37.5
66 years and older	15.6	25.8
*N*	16,655,799	140,557

^a^The numbers regarding the Dutch population regard insured between the age of 0 and 20

### Appendix 2: Different models to estimate healthcare expenses under supplementary health insurance

**Table 4:** Summary statistics of the different models tested to estimate the predicted claims for supplementary health insurance for individuals in year *t* − 1

		Mean expenses	Minimum expenses	Maximum expenses	Average expenses			*R* ^2^	Mean absolute prediction error
					1st tertile	2nd tertile	3rd tertile		
0	Actual healthcare expenses in year *t* − 1	221.04	0	21,010.59	0	100.12	566.37		
1	OLS	221.04	−25.13^a^	754.97	89.95	182.83	390.36	0.1874	199.06
2	GLM with gamma distribution and log link	222.68	36.57	1235.62	92.72	175.74	399.62	0.2078	199.89
3	GLM with normal distribution and log link	221.11	49.29	1023.97	97.10	176.53	389.76	0.1912	199.44
4	GLM with Poisson distribution and log link	221.04	46.47	985.46	95.68	176.14	391.36	0.1927	199.27

^a^Only 36 insured had negative predicted claims, which makes up for 0.033% of the data

Several models are tested to determine the predicted healthcare expenses for supplementary health insurance of individuals in year *t* based upon their background characteristics.

1. Ordinary least squares
2. Generalized linear model with a gamma distribution and a log link
3. Generalized linear model with a normal distribution and a log link.
4. Generalized linear model with a Poisson distribution and a log link.

Table 4 shows different summary statistics of all models. Since the differences regarding these statistics among the different models are minimal, we apply the most commonly recommended and used generalized linear model with a gamma distribution and a log link to determine the predicted claims for supplementary health insurance for each individual for each year.

## Footnotes

1 Van de Ven and Van Vliet [38] emphasize that, in case of adverse selection, asymmetric information is often used as a more general term for consumer information surplus. Consumer information surplus implies that individuals know their individual risk deviates from the average risk within their risk group (i.e., the risk group used by the insurer to differentiate the premium).

2 For empirical evidence on adverse selection see for instance: [11, 12, 14, 23, 25, 36, 48].

3 With premium differentiation, the premium for each insurance policy is adjusted to the individual’s risk [39]. As the differentiation is more refined, risk pools are less heterogeneous, implying less asymmetric information. Van de Ven and Van Vliet [38] for instance show that the use of risk factors like age, gender, health indicators, prior healthcare expenditures, supplementary insurance and region, reduces the consumer information surplus by about 80% in case of deductible choice.

4 This paper focuses on the potential of premium differentiation to counteract adverse selection. Another option would be to look at selective underwriting. However, due to the ‘guaranteed renewability’ clause in all Dutch supplementary insurances, selective underwriting for insured who currently have a supplementary insurance is very unlikely and therefore not a realistic simulation for the Dutch supplementary insurance market.

5 The insurance claims are corrected for inflation.

6 PCGs and DCGs are risk adjusters used as a proxy for health status based upon prior use of pharmaceuticals and prior hospital inpatient diagnoses, respectively [41].

7 Note that we select insured who have had the same supplementary insurance policy in the period 2006-2011 and that we use exactly these insured to simulate adverse selection. This might seem paradoxical since these insured did not opt out of the policy in the period 2006-2011. We consider this group of insured, however, as a pool of insured in which at a certain point in time the trend of adverse selection as observed in the Dutch supplementary health insurance occurs.

8 I.e., 2006 in the data is year *t*-2 in the simulations, 2007 is year *t* − 1, 2008 is year *t*, 2009 is year *t* + 1, 2010 is year *t* + 2, 2011 is year *t* + 3, 2007 is year *t* + 4, 2008 is year *t* + 5, 2009 is year *t* + 6, 2010 is year *t* + 7, 2011 is year *t* + 8, 2007 is year *t* + 9, etc.

9 Additionally, this looping process does not result in any problems concerning the background characteristics of insured when going from the year 2011 to 2007, because insured have the appropriate risk factors and corresponding predicted claims for each year, also when the looping process continues.

10 The average loading fee concerns the difference between the average annual reimbursed expenses under supplementary insurance and the average annual premium for supplementary insurance for the years 2008 to 2011 [44].

11 Note that the uptake is not 100%, as might be expected, since, in our simulations, the risk premium insured are willing to pay (i.e., €100 as the default option) is independent of the insured’s predicted claims while the loading fee (i.e., 23% of the average claims in a specific year) is dependent upon the average claims.

12 Note that if we would want to simulate the effect of risk attitude on the uptake of supplementary insurance in case of a continuation of adverse selection as observed in the Dutch supplementary health insurance (i.e., on average a 1% decrease in insurance uptake each year), we would not only need to adjust the risk premium the insured is willing to pay but also need to adjust the probability to opt out of the supplementary health insurance since the probability to opt out depends upon the assumptions regarding the insured’s risk attitude.
